# Evaluation de l’état vaccinal contre l’hépatite B et portage de l’Ag HBs chez les militaires Béninois en mission en Côte d’Ivoire

**DOI:** 10.11604/pamj.2019.32.19.16840

**Published:** 2019-01-11

**Authors:** Fanou Denis, Sehonou Jean, Vinasse Alfred, Agniwo Jérôme, Batcho Jimy, Etekpo Thomas, Tossou-Odjo Léon

**Affiliations:** 1 Service d’Hépatogastroentérologie CHU de Cocody, Abidjan, Côte d’Ivoire; 2 Médecine des Forces, Service de Santé des Armées, Cotonou, Bénin; 3 Service de Pathologie Digestive, Centre National Hospitalier et Universitaire Hubert Kutuku Maga, Cotonou, Bénin

**Keywords:** HVB, vaccination, séroprévalence, militaires, Benin, HVB, vaccination, seroprevalence, soldiers, Benin

## Abstract

**Introduction:**

Le but de cette étude est de déterminer le taux de couverture vaccinale anti-virus de l'hépatite B (anti-VHB) et la prévalence de l'AgHBs chez les soldats béninois en mission extérieure en Côte d'Ivoire.

**Méthodes:**

Cette étude transversale était réalisée au sein du bataillon béninois basé à Anankouakouté en Côte d'Ivoire. Les soldats de cette compagnie au décours d'une séance d'éducation sanitaire étaient soumis à un questionnaire sous forme d'interview. Il portait sur les données sociodémographiques, les facteurs de risques d'infection et les antécédents de vaccination contre l'hépatite B. Des prélèvements étaient faits à la recherche de l'Ag HBs et des anticorps antiHBs. En cas de positivité de l'Ag HBs, un complément de bilan était fait (alanines aminotransférase(ALAT), Ag HBe, AcantiHBe, AcAntiHBcIgM et ADN HBV par PCR).

**Résultats:**

Cent soixante-quinze militaires ont participé à cette étude (âge médian 31ans avec des extrêmes entre 23 et 52 ans; sex-ratio 5,73). Des taux protecteurs d'Ac Anti HBs étaient notés chez 41 militaires (23,4%). Cette immunité était post hépatitique B (25 cas/41) et post vaccinale (16 cas/41). Dix-huit militaires (10,3%) avaient une infection en cours par le VHB (Ag HBs+). L'infection était chronique (IgM anti HBc-et anticorps anti-HBc totaux + dans 18cas/18). Parmi les militaires infectés, 4 avaient une élévation des aminotransferases, 4 un Ag HBe positif et 4 une virémie élevée (ADN VHB >2000UI/ml).

**Conclusion:**

Le portage de l'AgHBs chez les militaires Béninois en mission est élevé. La couverture vaccinale est faible. Des stratégies d'intervention sont préconisées pour traiter ceux qui répondent aux critères et vacciner les non immunisés.

## Abstract (en)

**Introduction:**

This study aims to determine the rate of anti-hepatitis B virus coverage (anti-HBV) and the prevalence of Ag HBs among Beninese soldiers participating in military missions in Ivory Coast.

**Methods:**

We conducted a cross-sectional study within the Beninese battalion based in Anankouakouté in the Ivory Coast. The company’s soldiers were administered interview-based questionnaire during a health education session. It focused on socio-demographic data, risk factors for infection and a history of vaccination against hepatitis B. Samples were analyzed for Ag HBs and HBS antibodies. Sodiers positive for AgHBs underwent complementary tests (ALT, HBeAg, anti-HBe antibody, IgM anti-HBc antibody and HBV DNA using PCR).

**Results:**

One hundred seventy-five soldiers were enrolled in this study (with an average age of 31 years, ranging between 23 and 52 years; sex-ratio 5.73). Protective anti-HB antibody levels were found in 41 soldiers (23.4%). Some soldiers had had B hepatitis and had developed immunity to the virus (25 cases/41) while others had developed immunity after vaccination (16 cases/41). Eighteen soldiers (10.3%) had HBV (HBsAg+) infection. This infection was chronic (IgM anti-HBc - and anti-HBc antibody total + in 18 cases/18). Among the infected soldiers, 4 had elevated aminotransferase levels, 4 were found HBeAg-positive and 4 had elevated viremia levels (HBV DNA >2000IU/ml).

**Conclusion:**

The prevalence of Ag HBs among Beninese soldiers participating in military missions is high. However, immunization coverage is low. Intervention strategies should be implemented in order to treat subjects who meet the criteria and to vaccinate the unimmunized patients.

## Introduction

L'hépatite virale B est un problème mondial de santé publique, avec plus de 350 millions de porteurs chroniques pouvant transmettre le virus pendant des années [1]. Au Bénin, sa prévalence est estimée entre 6 et 8,6% [2-4]. La prévention par la vaccination est la meilleure stratégie pour éradiquer cette infection [5]. Au Bénin, seuls les enfants de six (06) semaines à onze (11) mois bénéficient gratuitement du vaccin dans le cadre du Programme Elargi de Vaccination (PEV) [6]. La vaccination des adultes relève de l'initiative individuelle. Le militaire en mission extérieure est une population à risque et les facteurs favorisants sont sa jeunesse (sujet sexuellement actif), sa grande mobilité et ses activités (gestion des catastrophes, ramassage des blessés, contact éventuel avec le sang lors des missions …). Au Bénin, il existe très peu de données concernant la prévalence de l'hépatite virale B et la vaccination des militaires. Le but de ce travail était de déterminer le taux de couverture vaccinale anti-virus de l'hépatite B (anti-VHB) et la prévalence de l'AgHBs chez les soldats Béninois en mission extérieure en Côte d'Ivoire.

## Méthodes

Il s'agissait d'une étude transversale, descriptive initiée au sein du bataillon Béninois basé à Anankouakouté en Côte d'Ivoire. La durée de l'étude était de 6 mois de novembre 2015 à avril 2016. Au décours d'une séance d'éducation sanitaire sur les hépatites virales B et C, tous les militaires béninois basés sur ce site ont été soumis à un questionnaire sous forme d'interview. Les variables suivantes étaient recueillies: 1) variables indépendantes: a) données sociodémographiques (âge-sexe-niveau d'instruction-situation matrimoniale); b) statut militaire (arme-ancienneté-catégorie); 2) facteurs de risque d'infection: a) les antécédents (transfusion, ictère, chirurgie, tatouage, scarification, hépatopathie familiale) et b) rapports sexuels non protégés avec un partenaire dont le statut n'était pas connu…); 3) l'état vaccinal contre l'hépatite virale B: le soldat était considéré comme vacciné s'il avait reçu les trois (03) doses de vaccin contre l'hépatite virale B. La vérification était faite sur le carnet de vaccination; le dépistage se faisait à l'Institut Pasteur basé au CHU de Cocody. Le dosage des trois marqueurs, par méthode ELISA (AgHBs- Ac anti HBc totaux et Ac anti HVC) se faisait à un coût forfaitaire dans le cadre d'une campagne nationale de dépistage.

Un hépato-gastroentérologue interprétait les résultats, complétait le bilan si nécessaire et proposait la vaccination aux sujet naïfs et/ ou non immunisés. Le contrôle de l'immunité par dosage quantitatif de l'anticorps anti-HBs se faisait chez les sujets vaccinés ou ayant éliminé le virus de l'hépatite B après un contact viral. Le sujet est immunisé lorsqu'il présente des titres protecteurs de l'anticorps anti HBs supérieurs à 10UI/l. Lorsque l'AgHBs était positif, un dosage de l'antigène HBe, de l'anticorps anti-HBe, de l'anticorps anti HBc de type IgM, des transaminases et de l'ADN viral par PCR en temps réel était demandé. La cytolyse hépatique était définie par des ALAT supérieurs à 40 UI/l. Le seuil de détection était de 20UI/ml pour le dosage de l'ADN viral B et le test utilisé était le « COBAS Ampliprep+ COBAS taq Man 48». Le reste du bilan se faisait dès le retour du soldat au Benin dans le cadre de la prise en charge. Le traitement des données a été effectué à l'aide du logiciel Epi info version 3.5.1. L'analyse a été descriptive par le calcul des moyennes et fréquences puis comparative des données à l'aide du KHI-deux (X2), du test de Fischer et de l'Odds ratio. Le seuil de signification p a été fixé à 5%. Pour chaque fréquence, un intervalle de confiance (IC) à 95 % a été calculé.

Aspects règlementaires et éthiques: une autorisation des autorités militaires était obtenue. Les participants étaient informés des avantages de l'intervention: possibilité de prise en charge en cas d'infection par le VHB, absence de répercussion de la découverte du statut sur l'aptitude à servir dans les Forces Armées Béninoises.

## Résultats

Au total, cent soixante-quinze militaires avaient participé à cette étude. La couverture vaccinale était de 9,1% et l'AgHBs était positif chez 18 des 175 militaires; soit une prévalence de 10,3%. Il n'était pas noté de co-infection ni avec le virus de l'hépatite C, ni avec le virus de l'immunodéficience humaine. Les caractéristiques sociodémographiques des participants sont colligées au niveau du Tableau 1.

**Tableau 1:** Caractéristiques sociodémographiques des participants

Paramètres	Effectifs (pourcentage)
**Sexe**	
Masculin	149 (85%)
Féminin	26 (15%)
**Niveau d’instruction**	
Primaire	12 (6,8%)
secondaire	124 (70,8%)
supérieur	39(22,4%)
**Catégorie de grade**	
officiers	20(11,4%)
sous-officiers	43 (24,5%)
militaires de rang	112 (35,9%)
**Situation matrimoniale**	
marié	142 (81,1%)
célibataire	33(18,9%)

**Vaccination contre l'hépatite virale B et mesure de l'efficacité vaccinale**: un total de 23,42% (n=41) des militaires étaient immunisés contre le virus de l'hépatite B. Parmi ces derniers, 14,3 % (n=25) étaient immunisés après un contact viral (Ag HBs négatif, Ac anti HBc totaux positifs) et 9,1% (n=16) avaient été vaccinés (3 doses de vaccin). La couverture vaccinale était donc de 9,1%. Les circonstances de vaccination étaient le départ en stage à l'extérieur dans 60% (n=10) où la vaccination était exigée dans le pays d'accueil, suivi de l'initiative personnelle dans 40% (n=6) des cas. La vaccination contre l'hépatite virale B par catégorie professionnelle était de 31,3% (n=5) pour les officiers, 43,7% (n=7) pour les sous-officiers et 25% (n=4) pour les militaires de rang avec une différence statistiquement significative (p=0,00016). Tous ceux qui avaient été vaccinés dans notre étude étaient immunisés (n=16).

**Caractéristiques biochimiques, sérologiques et virologiques des militaires antigène HBs positif**: les 18 militaires (3 officiers, 1 sous-officier et 14 militaires de rang) porteurs de l'Ag HBs avaient une infection chronique (Ag HBs +, Ac Anti HBc totaux +, Ac Anti HBc IgM -). L'AgHBe était négatif chez 77,8% et l'anticorps anti HBe était positif chez 22,2%. La moyenne des alanines aminotransférases (ALAT) était 26,16UI/l avec des extrêmes entre 7 et 74 UI/l. Une élévation était notée dans 4 cas; la charge virale ADN VHB était supérieure à 2000 UI/ml dans 4 cas (22,2%). Le Tableau 2 résume les caractéristiques biochimiques et virologiques des militaires porteurs de l'Ag HBs. Le Tableau 3 résume les facteurs de risque auxquels sont exposés les participants.

**Tableau 2:** Caractéristiques biochimiques et virologiques des 18 militaires ayant l’AgHBs positif

Paramètres	Effectifs (pourcentage)
**ALAT**	
Normal	14 (77,8%)
Elevé	4 (22,2%)
**ADN/VHB (UI/ml)**	
<20	3 (16,7%)
20-2000	11 (61,1%)
>2000	4 (22,2%)

**Tableau 3:** Facteurs de risque de transmission du virus de l’hépatite B

Facteurs de risque de transmission	AgHBs positif N=18	AgHBs négatif N=157	P	Significatif
**Rapports sexuels non protégés**	7	14	0,001	**S**
**Echanges de rasoirs**	2	2	0,008	**S**
**Ictère**	5	5	0,0002	**S**
**Hépatopathie familiale**	1	0	0,03	**S**
**Transfusion sanguine**	2	0	0,02	**S**
Scarifications	18	142	0,09	NS
Tatouages	0	6	0,36	NS
Soins dentaires	1	4	0,46	NS
Pédicure-manucure	1	2	0,18	NS

## Discussion

La couverture vaccinale anti-VHB était de 9,1% (n=16) chez les militaires en mission. Tous les soldats vaccinés sont immunisés dans notre étude. Dix d'entre eux (5,7%) avaient été en contact avec le virus et le dépistage n'avait pas été réalisé avant l'administration des vaccins. Cela pourrait s'expliquer par les circonstances de vaccination chez la plupart des soldats. Il s'agissait du départ en stage dans un pays où le vaccin était exigé (60%); dans 40% des cas de motivation personnelle sans un bilan initial. Ce taux est supérieur à celui retrouvé par Assi *et al.* chez les sapeurs-pompiers qui était de 2% en Côte d'Ivoire [7]. Dans les pays où la vaccination se faisait systématiquement chez les militaires, la couverture vaccinale variait entre 17,9% et 31,5% [8, 9]. Le vaccin contre le virus de l'hépatite B est efficace avec un pourcentage de séroconversion se situant entre 99 et 99,5% [10]. Cette efficacité a été confirmée dans notre étude où 100% des sujets vaccinés étaient immunisés; mais il se pose le problème de dépistage par une sérologie complète (Ag HBs, Ac anti HBc et Ac anti HBs) avant l'administration de ce vaccin. Une sensibilisation dans les garnisons et la formation continue des soignants pourront permettre de maîtriser cette insuffisance. Cependant le problème est d'ordre financier: le coût du dépistage (Ag HBs Ac Anti HBs et Ac Anti HBc est 46.200FCFA et soit 1,2 fois le salaire minimum interprofessionnel garanti qui était de 40.000FCFA).

Dans notre étude, la prévalence de l'Ag HBs était de 10,3%. Cette prévalence est supérieure à celles retrouvées dans les différentes études au sein de la population générale (8,35%) [3]; elle était de 5,2% chez les donneurs de sang [2] et de 8,6% au sein du personnel de santé au Bénin [4]. Elle est inférieure à celles trouvées en Côte d'Ivoire chez les sapeurs-pompiers par Assi *et al.* (13,11%) et chez les militaires donneurs de sang (12,5%) par Ehoussou *et al.* [7, 11]. Cette prévalence est largement supérieure à celles retrouvées chez les militaires en occident (France, Grèce) et en Nouvelle Calédonie variant entre 1,68 et 6,6% [8, 12, 13]. Ces différences peuvent s'expliquer par le fait que le Bénin est dans une zone de haute endémicité contrairement aux pays européens sus cités. A cette notion de haute endémicité, s'ajoute l'exposition de ces militaires à plusieurs facteurs de risque (rapports sexuels non protégés, échanges de rasoirs, ictère, hépatopathies familiales et transfusion) avec une différence statistiquement significative comme l'indiquait le Tableau 3. Tous les participants porteurs de l'Ag HBs étaient des porteurs chroniques: le statut de militaire en mission ne pourrait être incriminé comme étant un facteur de risque spécifique dans cette étude ; la transmission de l'HVB en Afrique Subsaharienne se fait le plus souvent par voie horizontale ou verticale. Enfin, la pratique de la scarification n'est pas retenue comme facteurs de risque dans la présente étude. Cette notion rejoint celle déjà avancée par certains auteurs Nigérians [14, 15]. D'autres études sont nécessaires pour confirmer ces notions et changer éventuellement de paradigmes quant aux modes de transmission spécifiques de l'HVB en Afrique subsaharienne.

## Conclusion

Le portage de l'Ag HBs chez les militaires Béninois en mission est de 10,3%. La couverture vaccinale est faible (9,1%). La vaccination systématique après le dépistage est recommandée. Cette étude préliminaire servira de base à d'autres investigations en matière de connaissance et de facteurs de risque de hépatopathies au sein de cette corporation au service de sa nation.

### Etat des connaissances actuelles sur le sujet

- L'hépatite virale B est un problème mondial de santé publique et le Bénin est une zone de haute endémicité;
- Cette maladie n'est pas bien documentée dans la population militaire africaine.

### Contribution de notre étude à la connaissance

- Notre étude montre une prévalence élevée de l'antigène HBs et une couverture vaccinale faible;
- Cette étude préliminaire servira de base à d'autres investigations en matière de connaissance et de facteurs de risque de hépatopathies au sein de cette corporation au service de la nation.

## Conflits d’intérêts

Les auteurs ne déclarent aucun conflit d’intérêts.

## Remerciements

Les auteurs remercient le Lieutenant-colonel Alotin Norbert, commandant le Bataillon Béninois en Côte d'Ivoire (et à travers lui tout le Haut Commandement Militaire) pour sa disponibilité et son implication personnelle dans l'organisation pratique des séances de sensibilisation de la troupe sur les hépatites virales.
